# ‘Women and babies are dying but not of Ebola’: the effect of the Ebola virus epidemic on the availability, uptake and outcomes of maternal and newborn health services in Sierra Leone

**DOI:** 10.1136/bmjgh-2016-000065

**Published:** 2016-10-07

**Authors:** Susan A Jones, Somasundari Gopalakrishnan, Charles A Ameh, Sarah White, Nynke R van den Broek

**Affiliations:** Centre for Maternal and Newborn Health, Liverpool School of Tropical Medicine, Liverpool, UK

## Abstract

**Background:**

We sought to determine the impact of the Ebola virus epidemic on the availability, uptake and outcome of routine maternity services in Sierra Leone.

**Methods:**

The number of antenatal and postnatal visits, institutional births, availability of emergency obstetric care (EmOC), maternal deaths and stillbirths were assessed by month, by districts and by level of healthcare for 10 months during, and 12 months prior to, the Ebola virus disease (EVD) epidemic. All healthcare facilities designated to provide comprehensive (n=13) or basic (n=67) EmOC across the 13 districts of Sierra Leone were included.

**Results:**

Preservice students were not deployed during the EVD epidemic. The number of healthcare providers in facilities remained constant (incidence rate ratio (IRR) 1.03, 95% CI 1.00 to 1.07). Availability of antibiotics, oxytocics, anticonvulsants, manual removal of placenta, removal of retained products of conception, blood transfusion and caesarean section were not affected by the EVD epidemic. Across Sierra Leone, following the onset of the EVD epidemic, there was a 18% decrease in the number of women attending for antenatal (IRR 0.82, 95% CI 0.79 to 0.84); 22% decrease in postnatal attendance (IRR 0.78, 95% CI 0.75 to 0.80) visits and 11% decrease in the number of women attending for birth at a healthcare facility (IRR 0.89, 95% CI 0.87 to 0.91). There was a corresponding 34% increase in the facility maternal mortality ratio (IRR 1.34, 95% CI 1.07 to 1.69) and 24% increase in the stillbirth rate (IRR 1.24, 95% CI 1.14 to 1.35).

**Conclusions:**

During the EVD epidemic, fewer pregnant women accessed healthcare. For those who did, an increase in maternal mortality and stillbirth was observed. In the post-Ebola phase, ‘readiness’ (or not) of the global partners for large-scale epidemics has been the focus of debate. The level of functioning of the health system with regard to ability to continue to provide high-quality effective routine care needs more attention.

**Key questions**

**What is already known about this topic?**

- Skilled birth attendance and emergency obstetric care are potentially life-saving care packages which need to be available 24/7 at health facility level to reduce maternal and newborn mortality.
- There is a lack of information regarding how routine health services are affected when large-scale severe epidemics occur.

**What are the new findings?**

- Across all districts in Sierra Leone, during the Ebola virus epidemic, there was an 18% decrease in the number of women attending for antenatal care, a 22% decrease in women seeking postnatal care and a 11% decrease in the number of women attending for birth at a healthcare facility.
- During the Ebola virus epidemic, there was a 34% increase in the facility maternal mortality ratio and 24% increase in the stillbirth rate.

**Recommendations for policy**

- In the post-Ebola phase, ‘readiness’ (or not) for future epidemics has been the focus of much debate. This ‘readiness’ is particularly important in fragile states where the impact of epidemics may be greater. Emergency preparedness plans need to be in place that take into account the capacity of healthcare facilities to provide both routine and emergency care as well as the need for early community mobilisation and involvement.

## Introduction

In May 2014, Sierra Leone, along with Guinea and Liberia, was hit by the biggest Ebola virus disease (EVD) epidemic ever recorded. The EVD epidemic was officially declared over in November 2015 by the Government of Sierra Leone and the WHO; there had been 8704 cases and 3589 deaths.^1^ ^2^ However, there was a flare up of EVD reported in Sierra Leone in January 2016 and declaration that the country was again free of EVD on 17 March 2016.^3^ It is considered likely that the full impact of the EVD outbreak is not just related to the disease itself but also to its effect on other aspects of healthcare provision. This might be particularly so for maternity services as these are expected to be available 24/7 at health facility level. At least 80% of all maternal deaths globally result from five complications which are well understood and can be prevented or managed by experienced healthcare providers. Key services that need to be in place include antenatal (ANC) and postnatal care (PNC), skilled birth attendance (SBA) and, for the 15% of women who are expected to have a complication, access to and availability of, either basic (BEmOC) or comprehensive emergency obstetric care (CEmOC; table 1).^4^

**Table 1:** Signal functions of EmOC^17^

BEmOC	CEmOC
1. iv/im antibiotics	All included in BEmOC (1–7) plus
2. iv/im oxytocic drugs	8. Blood transfusion
3. iv/im anticonvulsants	9. Caesarean section
4. Manual removal of retained placenta	
5. Removal of retained products of conception (by manual vacuum aspiration)	
6. Assisted vaginal delivery (ventouse delivery)	
7. Resuscitation of the newborn (using a bag and mask)	

BEmOC, basic emergency obstetric care; CEmOC, comprehensive emergency obstetric care; EmOC, emergency obstetric care; im, intramuscular; iv, intravenous.

It can be difficult to differentiate between complications of pregnancy such as obstetric haemorrhage (the leading cause of maternal death in low resource settings) and the case definition of EVD, which could affect patient management.^5^ Women suspected of having EVD would normally be isolated until their Ebola status is confirmed. However, such isolation may also prevent women from receiving timely obstetric care.^6^ Obstetric care is considered one of the most high-risk areas for exposure to body fluids, through which EVD is spread, and as a consequence healthcare providers face increased risks of exposure to Ebola and so may be reluctant to assist at the time of birth or carry out invasive procedures if adequate protection is not in place.

In October 2014, the United Nations Population Fund (UNFPA) estimated that 800 000 women were due to give birth over the following 12 months in the three EVD affected countries and that 12 000 women and babies would require some level of emergency obstetric care (EmOC).^7^ Before the onset of the EVD epidemic, Sierra Leone had made excellent progress towards achieving Millennium Development Goal 5 with an estimated 52% reduction in the maternal mortality ratio between 1990 and 2013 and 97% of women attending for one or more ANC visits.^8^ However, with a still fragile health system and an estimated maternal mortality ratio of 1100/100 000 live births, Sierra Leone could ill afford to lose the gains it had made.^9^

Although there has been significant interest in, and critique of, the international response to the EVD epidemic,^10^ much less is known about ‘readiness’ and functioning of the existing health system for non-EVD-related and more routine health service provision which, it could be argued, is at least as important during an epidemic. This study aimed to look at the impact of the EVD epidemic on the availability, uptake and outcomes of maternal and newborn health services in Sierra Leone.

## Methods

### Selection of healthcare facilities

Sierra Leone is divided into 13 districts, each district has one healthcare facility designated to provide CEmOC and five or six designated to provide BEmOC. During the epidemic, there was concern about the impact of Ebola on pregnant women, in particular the difficultly in differential diagnosis between obstetric emergencies and infection with the Ebola virus^6^ and the increased risk to healthcare workers because of the high exposure to body fluids during obstetric care. The decision to focus on provision and uptake of care in BEmOC and CEmOC facilities was based on their key role in providing EmOC. Data were unavailable from two BEmOC facilities (one each in Bo and Kenema districts) as contact with these facilities could not be established. Thus, we included all 13 facilities designated to provide CEmOC across Sierra Leone and 65 of 67 facilities designated to provide BEmOC. All included healthcare facilities are designated to provide ANC, SBA and PNC.

### Data collection

The numbers of EVD cases and peak incidence of the disease varied over time across the 13 districts of Sierra Leone, with some districts reporting higher overall numbers than others and a corresponding difference in facility impact. Therefore, we collected data on the number of EVD cases per week per district from confirmed patient databases and situation reports from the National Ebola Response Centre (http://nerc.sl/).

An electronic data collection tool developed at the Centre for Maternal and Newborn Health—Liverpool School of Tropical Medicine (LSTM) was used to aid data collection and possibly reduce the risk of any cross-infection. Data were collected by experienced LSTM Sierra Leonean technical officers based in Freetown who had worked within the health system and understood the impact Ebola was having on the country. All data collectors were given instructions on how to maintain their own safety when visiting facilities and where allowed to restrict their visits if they felt their own safety was at risk. Data collectors reported any difficulties in data collection due to the epidemic and a collective decision was made whether to suspend or delay data collection dependent on conditions within the each facility. Data were able to be gathered from all but two of the targeted health facilities.

Data were obtained on the availability of healthcare providers, availability and provision of EmOC signal functions, drugs, equipment, number of ANC and PNC visits, number of births at the facility, reported number of emergency complications, maternal deaths, and stillbirths.^11^ Signal functions were classified as being available if the equipment, drugs and appropriately trained staff were available to perform the signal function. Data for the availability of healthcare providers was obtained from facility attendance registers. Data regarding availability (or not) of equipment and drugs was obtained from facility registers.

Information on each indicator of interest was obtained retrospectively from facility registers for each month (for each of the 12 months before and 10 months during the EVD epidemic) during a visit made to each health facility for this purpose by trained staff based in Freetown.

### Data analysis

For each facility, data for each month was available and analysed for four groups of outcomes: (1) number of staff available by cadre; (2) availability of each EmOC signal function; (3) number of antenatal visits, postnatal visits and births; (4) maternal deaths and stillbirths.

To examine the possible effects of the EVD epidemic on each of these outcomes, mixed-effects models were used because of the longitudinal nature of the data. To assess the impact of the EVD epidemic on each of the outcomes of interest, an indicator variable was defined (occurrence of one or more EVD cases in district in the immediately preceding month). Relevant risk factors were: type of facility (BEmOC or CEmOC), district and month of the year; each of these was defined as a categorical variable. For all analyses, facilities were treated as random effects.

An alternative approach to account for the EVD epidemic used the occurrence of EVD cases in any preceding month. This approach did not yield different results unless indicated in the Results section. A further alternative approach would be to use the first occurrence of EVD in the country. This approach was not considered as it would be less sensitive to the likely variation within districts and between healthcare facilities.

p Values were obtained using likelihood ratio tests, and when this was not possible (because fitting of the submodel with Ebola excluded was not possible), Wald tests are reported. A p value <0.05 was considered to be statistically significant. Tests of interaction between facility type and EVD were performed using models which involved only the explanatory variables facility type and EVD presence.

#### For availability of healthcare providers

Mixed-effects Poisson regression models were used, to model separately the effect of the EVD epidemic on the total number of staff and the number of staff in each cadre. Poisson models were used because of the count nature of the data. Type of facility and district were both included. Separate district-level analyses were not performed since most healthcare providers are drawn from a central pool and a population of limited size. Means and ratios are reported for occurrences of Ebola and type of facility.

#### For availability of EmOC

For availability of signal functions (a binary outcome, available or not available), mixed-effects logistic regression models were used. For each month, the outcome was whether the signal function was in principle available or not. ORs are reported for the association of availability of each signal function with onset of the EVD epidemic after accounting for variables within the analysis. District was included for signal functions 1, 2 and 3; type of facility for signal functions 1, 2, 3 and 7 (otherwise they were omitted to ensure plausibility of the model); month was included for all signal functions.

For each facility assessment, availability (or not) of the required cadre of healthcare provider, equipment and/or drugs needed for each of the signal functions of EmOC was assessed.

#### For uptake of services

For numbers of events (ANC visits, PNC visits and births) mixed-effects Poisson regression models were used. Type of facility, month and district were each included in analysis of data for all districts. For analysis of each district, type of facility and month were included. Ratios of mean number of events per facility are reported for onset of EVD epidemic and type of facility.

#### Maternal deaths and stillbirths

The stillbirth rate was calculated for each facility as number of stillbirths recorded per 1000 live births. The maternal mortality ratio was calculated as number of maternal deaths recorded per live 100 000 births.

Mixed-effects Poisson regression models were used, with number of live births used to define exposure, and thus derive incidence rates. Type of facility, district and month were included in the analysis of data for all districts. For analysis of each district, facility was included when there were deaths for both types of facility (CEmOC and BEmOC), otherwise only data for the type of facility at which deaths occurred were used. Incidence rate ratios (IRRs) are reported for EVD onset (or not) and for type of facility (when included).

The statistical package Stata V.12.1 was used for all analyses.

### Ethical approval

Ethical approval for the study was obtained from the Sierra Leone Research and Scientific Committee and from the LSTM Ethics Committee (reference number 15.004RS).

## Results

**Figure 1:**
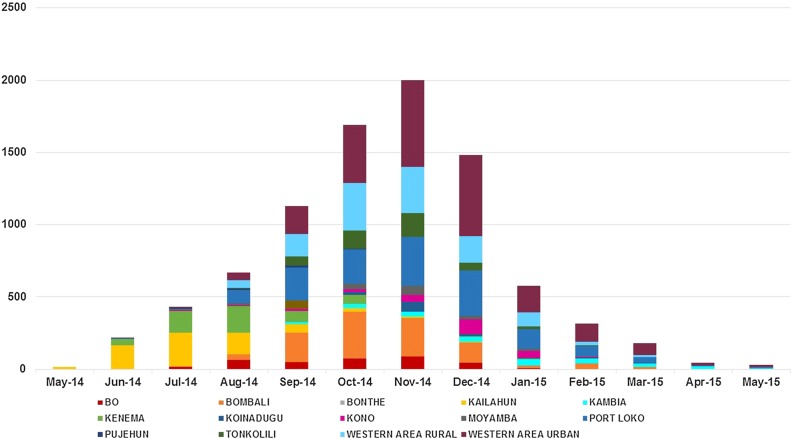
Number of Ebola Virus cases in Sierra Leone, number of antenatal visits (ANC), postnatal care (PNC) visits and births at health facility level (institutional delivery).

Data for a total 78 facilities were available for 22 months, giving a total of 1716 month–facility combinations of which 474 with EVD and 1242 with no EVD present. All districts had at least 4 months in which EVD cases were reported (figure 1).

### Availability of healthcare providers

Overall, there was a small 3% (IRR 1.03, 95% CI 1.00 to 1.07; p=0.09), but not statistically significant increase in the total number of healthcare providers deployed and working at both CEmOC-level and BEmOC-level health facilities (excluding students and traditional birth attendants) following the onset of the EVD epidemic (table 2). However, the number of student healthcare providers decreased by two-thirds (IRR 0.33, 95% CI 0.29 to 0.37; p<0.001). When combined, the total (including trainees) number of healthcare providers in place reduced by 8% (IRR 0.92, 95% CI 0.90 to 0.95).

**Table 2:** Association between the EVD epidemic and numbers and cadres of healthcare providers in post in Sierra Leone at healthcare facilities designated to provide BEmOC or CEmOC

	Mean number of healthcare providers per healthcare facility				EVD epidemic*	
	Facility providing BEmOC		Facility providing CEmOC			
Cadre of staff	No Ebola (n=1035)	Ebola (n=395)	No Ebola (n=207)	Ebola (n=79)	Ratio of means (95% CI)	p Value
Specialist doctor	0	0	0.34	0.39	1.00 (0.67 to 1.51)	0.99
Medical doctor‡	0.002	0.018	1.61	1.90	1.12 (0.93 to 1.36)	0.24
CHO	1.05	1.10	1.29	1.46	1.03 (0.93 to 1.14)	0.54
Registered midwives	1.04	1.02	2.63	2.71	0.97 (0.88 to 1.06)	0.52
Registered nurses	0	0	0.55	0.60	0.98 (0.71 to 1.37)	0.92
Nurse anaesthetist			2.10	2.40	1.01 (0.86 to 1.22)	0.87
State enrolled community nurses	1.56	1.85	9.96	11.33	1.06 (1.00 to 1.12)	0.06
Maternal and child health aides	1.82	2.04	1.52	1.51	1.02 (0.94 to 1.10)	0.65
Nurse aide	0.35	0.42	2.57	2.69	1.03 (0.91 to 1.16)	0.62
Total of cadre above	5.82	6.46	24.0	26.2	1.03 (1.00 to 1.07)	0.09
Traditional birth attendant	3.85	4.35	1.19	0.98	1.03 (0.97 to 1.09)	0.33
Student§	1.86	0.34	5.28	3.39	0.33 (0.29 to 0.37)	<0.001
Total	11.49	11.11	29.2	29.9	0.92 (0.90 to 0.95)	<0.001

*Ebola cases were confirmed in the district in the previous month.

‡Data missing at one CEmOC facility from March 2014, treated as 0 in derivation of totals, but missing in analysis of this cadre.

§Data missing throughout at five facilities, and on four other occasions in April and May 2013, treated as 0 in derivation of totals, but missing in analysis of this cadre.

BEmOC, basic emergency obstetric care; CEmOC, comprehensive emergency obstetric care; CHO, Community Health Worker; EVD, Ebola virus disease.

### Availability of signal functions of EmOC

Overall, for all districts combined, there is no evidence of an association between the onset of EVD epidemic in that district and the ability (or not) to provide the components (signal functions) of the EmOC care package (table 3). This includes intravenous or intramuscular antibiotics, oxytocics, anticonvulsants, manual removal of a retained placenta, removal of retained products of conception (signal functions 1–5), blood transfusion and caesarean section (signal functions 8 and 9).

**Table 3:** Association between EVD epidemic and availability of signal functions at healthcare facilities designated to provide BEmOC or CEmOC

	Availability of EmOC signal function (% of month–facility occasions when signal function available)						Effect of EVD epidemic	
	Facility providing BEmOC		Facility providing CEmOC		All facilities combined			
EmOC signal function	No Ebola (n=1035)	Ebola (n=395)	No Ebola (n=207)	Ebola (n=79)	No Ebola (n=1242)	Ebola (n=474)	OR (95% CI)	p Value
1. im/iv antibiotics	95.7	96.5	95.7	93.7	95.7	96.0	1.01 (0.48 to 2.12)	0.99
2. im/iv oxytocics	94.1	91.7	95.2	92.4	94.3	91.8	0.67 (0.39 to 1.16)	0.59
3. im/iv anticonvulsants	97.6	98.0	95.7	96.2	97.3	97.7	1.99 (0.71 to 5.57)	0.18
4. Manual removal of placenta	100.0	100.0	100.0	100.0	100.0	100.0	NV	
5. Removal of retained products of conception	79.7	84.3	100.0	100.0	83.1	86.9	1.65 (0.71 to 3.83)	0.23
6. Assisted vaginal delivery	84.4	81.3	100.0	100.0	87.0	84.4	0.45 (0.20 to 1.03)	0.056
7. Neonatal resuscitation	79.8	88.4	77.8	81.0	79.5	87.1	NR	
8. Blood transfusion	NA	NA	91.3	86.1	NA	NA	NE	
9. Caesarean section	NA	NA	91.6	91.6	NA	NA	NV	

BEmOC, basic emergency obstetric care; CEmOC, comprehensive emergency obstetric care; EmOC, emergency obstetric care; EVD, Ebola virus disease; im, intramuscular; iv, intravenous; n, number of facility–month combinations; NA, not applicable, signal function not expected at BEmOC facilities; NE, not estimable: in one district the initiation of availability of this signal function coincided with Ebola, in all others there is no variation over months; NV, no variation over time, within each facility, regarding availability of signal function.

When the analysis treated month–district combinations with no cases of EVD reported as EVD-free months, there was no difference in any of the signal functions except for ability to perform neonatal resuscitation (signal function 7) which increased at both BEmOC and CEmOC level. At both levels, this was attributed to increased availability of equipment required, unrelated to the EVD epidemic, that is, bag and masks for resuscitation.

Assisted vaginal delivery (signal function 6) was always available at facilities designated to provide CEmOC during the EVD epidemic. When only BEmOC-level facilities were considered, the estimated OR was 0.45 (95% CI 0.20 to 1.03).

### Factors affecting availability of signal functions of EmOC

Overall, the numbers and cadres of healthcare provider were noted to be in post for each of the signal functions of EmOC across all districts before and after the EVD epidemic, and this was not reported to be a limiting factor for EmOC availability. However, equipment and/or drugs were not always available. Where intravenous/intramuscular antibiotics were not available (72/1716 occasions among 10 facilities), this was equally likely to be due to lack of drugs (42/72 occasions) or lack of equipment (syringes or needles; 41/72 occasions). Non-availability of intravenous/intramuscular oxytocics (110/1716 occasions among 17 facilities) was more commonly due to lack of oxytocics (any type; 87/110 occasions) than equipment (syringes or needles; 40/110 occasions). Non-availability of anticonvulsants (45/1716 occasions among 10 facilities) was because of lack of an anticonvulsant (magnesium sulfate or diazepam; 4/45 occasions) and equipment (syringes or needles; 41/45 occasions).

Blood transfusion was available in all but one CEmOC (lack of equipment; cross matching reagents, blood storage refrigerator). Caesarean section was available at 12 out of 13 CEmOC facilities. There were 24 month–facility combinations when it was not available—all 22 months in one facility due to lack of an operating theatre and the first 2 months due to lack of qualified staff.

### Uptake of services

**Figure 2:**
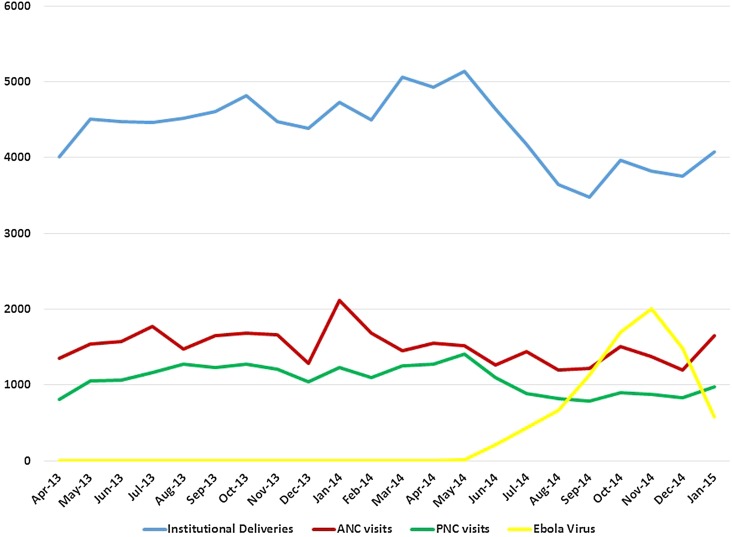
Number of Confirmed Ebola Virus (EBV) cases by district from time of onset of epidemic across Sierra Leone.

Data for ANC and PNC visits were available for eight districts of which six recorded a statistically significant decrease in the number of ANC and PNC visits during the Ebola epidemic (table 4 and figure 2).

**Table 4:** Association between EVD, number of women attending for ANC, PNC and delivery at a healthcare facility; overall and by district disaggregated by level of healthcare (CEmOC or BEmOC)

	ANC visits			PNC visits			Facility births		
	CEmOC vs BEmOC	Ebola		CEmOC vs BEmOC	Ebola		CEmOC vs BEmOC	Ebola	
District	Ratio (95% CI)	Ratio (95% CI)	p Value	Ratio (95% CI)	Ratio (95% CI)	p Value	Ratio (95% CI)	Ratio (95% CI)	p Value
Bo (n=130)	3.72 (0.90 to 15.4)	0.76 (0.71 to 0.81)	<0.001	1.52 (0.63 to 3.67)	0.78 (0.72 to 0.85)	<0.001	3.09 (1.25 to 7.62)	1.15 (1.07 to 1.24)	<0.001
Bombali (n=88)	NA	1.04 (0.89 to 1.20)	0.62	NA	1.04 (0.92 to 1.19)	0.53	9.00 (4.70 to 17.1)	0.78 (0.72 to 0.83)	<0.001
Bonthe (n=0)	ND	ND		ND	ND		1.66 (1.04 to 2.67)	0.76 (0.64 to 0.91)	0.003
Kailahun (n=110)	1.89 (1.64 to 2.18)	0.83 (0.78 to 0.90)	<0.001	1.49 (0.97 to 2.31)	0.87 (0.80 to 0.94)	<0.001	1.50 (0.99 to 2.27)	0.75 (0.70 to 0.81)	<0.001
Kambia (n=126)	1.09 (0.82 to 1.44)	0.69 (0.62 to 0.77)	<0.001	1.01 (0.51 to 1.97)	0.78 (0.67 to 0.90)	<0.001	3.11 (2.45 to 3.95)	0.60 (0.53 to 0.67)	<0.001
Kenema (n=44)	5.94 (5.46 to 6.46)	0.82 (0.76 to 0.88)	<0.001	4.80 (4.41 to 5.22)	0.71 (0.65 to 0.78)	<0.001	6.47 (2.63 to 15.90)	0.92 (0.86 to 0.98)	0.013
Koinadugu (n=0)	ND	ND		ND	ND		5.34 (3.40 to 8.37)	0.81 (0.74 to 0.90) 0.93 (0.82 to 1.06)	<0.001 0.27
Kono (n=131)	9.8 (2.6 to 367)	0.74 (0.69 to 0.80)	<0.001	3.01 (1.06 to 8.58)	0.53 (0.48 to 0.59)	<0.001	3.47 (1.77 to 6.81)	0.87 (0.79 to 0.96)	0.006
Moyamba (n=0)	ND	ND		ND	ND		0.98 (0.66 to 1.44)	0.83 (0.76 to 0.90)	<0.001
Port Loko (n=101)	NA	0.87 (0.80 to 0.93)	<0.001	NA	0.65 (0.58 to 0.72)	<0.001	1.19 (0.72 to 1.94)	0.58 (0.52 to 0.64)	<0.001
Pujehun (n=0)	ND	ND		ND	ND		1.03 (0.60 to 1.76)	0.88 (0.80 to 0.98)	0.014
Tonkolili (n=100)	NA	0.92 (0.84 to 1.01)	0.09	NA	1.02 (0.90 to 1.15)	0.78	2.59 (1.31 to 5.12)	0.98 (0.89 to 1.08)	0.73
Western area (n=0)	ND	ND		ND	ND		11.03 (2.95 to 41.2)	0.99 (0.94 to 1.03)	0.51
All	3.10 (1.88 to 5.11)	0.82 (0.79 to 0.84)	<0.001	1.77 (1.16 to 2.68)	0.78 (0.75 to 0.80)	<0.001	2.72 (2.10 to 3.52)	0.89(0.87 to 0.91)	<0.001

ANC, antenatal care; BEmOC, basic emergency obstetric care; CEmOC, comprehensive emergency obstetric care; EVD, Ebola virus disease; n, number of facility–month combinations; NA, not applicable (no data for the CEmOC in this district); ND, no data available; PNC, postnatal care.

Overall, for all districts combined, there was a statistically significant reduction in the numbers of ANC and PNC visits after the onset of the EVD epidemic. The estimated reduction for ANC visits was 18% (IRR 0.82, 95% CI 0.79 to 0.84; p<0.001) and for PNC visits was 22% (IRR 0.78, 95% CI 0.75 to 0.80; p<0.001).

For both ANC and PNC visits, there were statistically significant differences (G2=29.6, df=1, p<0.001 and G2=6.51, df=1, p=0.01, respectively) between facility type in the IRRs for the impact of the EVD epidemic with greater reductions at CEmOC level than at BEmOC level. For ANC visits at BEmOCs, there was a 14% decrease (IRR 0.86, 95% CI 0.83 to 0.89); whereas for CEmOCs, there was a 25% decrease (IRR 0.75, 95% CI 0.72 to 0.78). For PNC visits, there were 20% and 27% decreases at BEmOCs and CEmOCs, respectively (IRR 0.80, 95% CI 0.77 to 0.83 and IRR 0.73, 95% CI 0.69 to 0.77).

ANC and PNC data were not available for the CEmOC-level facility in two of the districts; ANC and PNC data were not available in four districts.

The number of deliveries that occurred at health facility level (data available for all 13 districts) also showed a statistically significant decrease of 11% (IRR 0.89, 95% CI 0.87 to 0.91). There was a statistically significant difference (G2=11.4, df=1, p=0.0007) between facility type in the IRRs for the impact of the EVD epidemic. For BEmOCs, there was a 14% decrease (IRR 0.86, 95% CI 0.84 to 0.89); whereas for CEmOCs, there was an 8% decrease (IRR 0.92, 95% CI 0.89 to 0.95). Bo was the only district to report an increase in number of deliveries following the start of the EVD epidemic, with a statistically significant increase in numbers occurring mainly at CEmOC level (IRR 1.15, 95% CI 1.07 to 1.24; p<0.001).

The decrease in number of deliveries at CEmOC level was associated with an overall increase in the caesarean section rate (number of caesarean sections per number of facility births) of 14% (IRR 1.14, 95% CI 1.06 to 1.22) confirming this procedure continued to be available.

### Maternal mortality ratio and stillbirth rate

A total of 464 maternal deaths and 55 095 live births were recorded at healthcare facility level between 1 May 2013 and 31 January 2015; 152 maternal deaths were recorded during the EVD epidemic months and 312 in the 12 months when no EVD was reported in the previous month.

For all districts combined, the facility-based maternal mortality ratio increased by 34% after onset of the EVD epidemic (IRR 1.34, 95% CI 1.07 to 1.69; table 5). When type of facility was considered separately (CEmOC or BEmOC), the increase in maternal deaths was significant at CEmOCs level (p<0.001) but not at BEmOC level (p=0.35). However, the interaction between facility type and onset of the EVD epidemic was not statistically significant (G2=2.13, df=2, p=0.35).

**Table 5:** Association between EVD epidemic and facility MMR overall and by district disaggregated by level of healthcare (CEmOC or BEmOC)

				MMR (maternal deaths/100 000 live births)							
				Facility providing CEmOC		Facility providing BEmOC		Comparison of CEmOC vs BEmOC		Comparison of Ebola vs no Ebola	
Type of facility or district	Number of months with Ebola cases	Number of maternal deaths	Number of live births	No Ebola (n=201)	Ebola (n=78)	No Ebola (n=1034)	Ebola (n=390)	IRR (95% CI)	p Value	IRR (95% CI)	p Value
All facilities				1962	3097	59	55	42 (26 to 69)	<0.001	1.34 (1.07 to 1.69)	0.01
BEmOCs										0.64 (0.18 to 2.24)	0.53
CEmOCs										1.48 (1.21 to 1.81)	<0.001
Bo (n=132)	7	54	4389	2638	5519	53	0	93 (13 to 673)	<0.001	2.2 (1.3 to 3.8)	0.004
Bombali (n=22)	7	51	4812	1634	1850	0	0	ND		1.2 (0.6 to 2.2)	0.58
Bonthe (n=0)	4	1	1429	0	0	0	455	Only 1 event in this district			
Kailahun (n=131)	8	13	4233	1687	1266	0	122	43 (6 to 331)	<0.001	1.1 (0.3 to 3.6)	0.86
Kambia (n=132)	4	38	3384	2800	11 935	58	0	68 (9 to 495)	<0.0001	2.1 (0.8 to 5.3)	0.17
Kenema (n=22)	7	48	5431	1839	1324	0	0	ND		0.7 (0.4 to 1.5)	0.37
Koinadugu (n=132)	4	35	3258	2120	2323	141	0	17 (4 to 70)	<0.001	1.1 (0.4 to 2.6)	0.87
Kono (n=131)	7	8	2620	708	332	142	0	5.5 (1.1 to 27)	0.05	0.3 (0.04 to 2.6)	0.23
Moyamba (n=132)	7	16	3227	2987	1242	191	220	24 (7 to 85)	<0.001	0.7 (0.2 to 2.5)	0.57
Port Loko (n=22)	7	13	2774	3709	2857	0	0	ND		1.1 (0.2 to 4.9)	0.91
Pujehun (n=21)	5	14	3462	2480	4.697	0	0	ND		1.9 (0.6 to 5.5)	0.29
Tonkolili (n=126)	5	29	3221	1122	8796	102	0	38 (8.9 to 160)	<0.001	4.5 (2.0 to 10.0)	<0.001
Western area (n=130)	7	143	12 855	1550	2500	78	0	22 (8 to 60)	<0.001	1.5 (1.0 to 2.0)	0.03

BEmOC, basic emergency obstetric care; CEmOC, comprehensive emergency obstetric care; EVD, Ebola virus disease; IRR, incidence rate ratio; MMR, maternal mortality ratio; n, number of facility–month combinations; ND, no deaths reported for one level.

The total number of reported stillbirths was 3589 giving an overall facility-based stillbirth rate of 60.5 per 1000 births. Overall, there was a 24% increase in the incidence of stillbirth (IRR 1.24, 95% CI 1.14 to 1.35; table 6). However, there was an interaction between type of facility and onset of the EVD epidemic (G2=15.6, df=2, p<0.001). When type of facility was considered separately, the increase was significant at CEmOC level (IRR 1.27, 95% CI 1.16 to 1.39; p<0.001) but not at BEmOC level (IRR 1.07, 95% CI 0.85 to 1.39; p=0.57).

**Table 6:** Association between onset of EVD epidemic and facility SBR overall and by district disaggregated by level of healthcare (CEmOC or BEmOC)

				Mean incidence of stillbirths (per 1000 births)							
				Facility providing CEmOC		Facility providing BEmOC		Comparison of CEmOC vs BEmOC		Comparison of Ebola vs no Ebola	
Type of facility or district	Number of months with Ebola	Total number of stillbirths	Total number of births in district	No Ebola (n=201)	Ebola (n=78)	No Ebola (n=1034)	Ebola (n=390)	IRR (95% CI)	p Value	IRR (95% CI)	p Value
All facilities				138	194	18	17	13.9 (8.7 to 22.9)	<0.001	1.24 (1.14 to 1.35)	<0.001
Facility providing BEmOC										1.07 (0.85 to 1.35)	0.57
Facility providing CEmOC										1.27 (1.16 to 1.39)	<0.001
Bo (n=132)	7	161	4550	68	141	15	13	8.13 (1.8 to 37)	0.001	1.67 (1.22 to 2.28)	0.001
Bombali (n=131)	7	341	5153	105	134	13	8	12.1 (2.9 to 50)	0.001	1.21 (0.97 to 1.54)	0.10
Bonthe (n=132)	4	28	1459	41	17	15	24	3.6 (1.7 to 7.6)	0.001	1.05 (0.4 to 3.0)	0.93
Kailahun (n=131)	8	179	4409	124	94	21	26	5.5 (2.0 to 15.6)	0.001	1.0 (0.7 to 1.4)	0.97
Kambia (n=132)	4	173	3558	120	108	19	16	6.5 (4.4 to 9.5)	<0.001	0.9 (0.5 to 1.6)	0.72
Kenema (n=131)	7	369	5802	112	148	15	20	9.8 (3.4 to 28.7)	<0.001	1.3 (1.1 to 1.6)	0.01
Koinadugu (n=132)	4	190	3448	88	94	39	34	3.0 (2.1 to 4.2)	<0.001	1.0 (0.7 to 1.5)	0.88
Kono (n=131)	7	206	2826	197	242	11	8	16.3 (9.9 to 26.7)	<0.001	1.2 (0.9 to 1.5)	0.33
Moyamba (n=132)	7	91	3318	128	245	11	8	30.3 (2.3 to 394)	0.009	1.4 (0.9 to 2.2)	0.13
Port Loko (n=132)	7	177	2951	382	453	13	21	25.9 (6 to 105)	<0.001	1.2 (0.8 to 1.7)	0.38
Pujehun (n=131)	5	80	3542	168	162	1	0	398 (55 to 2858)	<0.001	1.1 (0.7 to 1.8)	0.73
Tonkolili (n=126)	5	211	3426	137	410	30	27	11.9 (0.9 to 152)	0.06	1.9 (1.4 to 2.4)	<0.001
Western area (n=130)	7	1386	14 240	155	183	27	28	7.9 (1.1 to 59)	0.04	1.1 (1.0 to 1.2)	0.06

BEmOC, basic emergency obstetric care; CEmOC, comprehensive emergency obstetric care; EVD, Ebola virus disease; IRR, incidence rate ratio; n, number of facility–month combinations; SBR, stillbirth rate.

## Discussion

### Main findings

Across Sierra Leone, following the onset of the EVD epidemic, there was a decrease of 18% in the number of women attending for ANC, 22% decrease in attendance for PNC and an 11% decrease in the number of women attending for birth at a healthcare facility able to provide emergency obstetric and newborn care. For women who did access care, there was a corresponding statistically significant 34% increase in the facility maternal mortality ratio and 24% increase in the stillbirth rate.

### Strengths and weaknesses

To the best of our knowledge, this is the only study to have collected data on maternity services uptake and outcomes across all the 13 districts of Sierra Leone and including time both before and during the EVD epidemic. Routinely collected data were obtained from registers at each healthcare facility retrospectively. The EVD epidemic was unexpected with regard to severity and length, and therefore it was not possible to design a prospective study. This was an example of operations research and use of routine data. We were unable to a priori strengthen the data or data collection and recording systems before the study took place.

Staffing levels required for each type of facility are provided by the Government of Sierra Leone in the Basic Package of Essential Health Services;^12^ however, there are ongoing shortages of staff. This study did not report if healthcare facilities met the prescribed cadre and numbers of healthcare providers but rather on change, or not, in the number in post during the EVD epidemic. The study did not assess the quality of care provided; for example, there was no assessment of the timeliness with which interventions were provided and whether or not these were provided too late in some cases to be live-saving. Similarly, we did not assess the quality of resuscitation of the newborn to determine if preventable stillbirths occurred as a result of poor or non-resuscitation efforts at the time of birth.

The analysis is defined by reference to the onset (or not) of EVD in any particular district. We did not have access to village-level data which may have provided a more detailed pattern and analysis regarding availability and uptake of care. All healthcare facilities designated to provide EmOC were included in the study but it is conceivable that ANC, PNC or skilled attendance at birth can also be provided at lower level healthcare facilities which were not included in this study.

### Interpretation in light of other studies

Anecdotal reports at the start of the epidemic mentioned healthcare providers ‘leaving their posts’ and ‘refusing to provide patient care’. This study shows that staff did remain in post. The exception was student preservice attachments, reflecting the countrywide closure of schools during the EVD epidemic.^13^

SBA, ANC and PNC continued to be available, and the EVD epidemic did not lead to a decrease in availability of EmOC. However, assisted vaginal delivery (ventouse) was not available across all healthcare facilities designated to provide BEmOC. There were also differences with regard to uptake of care; at hospital or CEmOC levels, the decrease in number of women accessing for ANC or PNC suggested that perhaps women only attended larger healthcare facilities in more urban and populated areas if they had to do so for an emergency and might have attended as late as possible.

It is likely that fear of contracting Ebola during visits to healthcare facilities and (unsubstantiated) rumours such as that healthcare providers were injecting patients with the virus, led women to stay away and/or access care late. Perceptions about the quality of patient care by the public during the epidemic may also have reduced numbers attending at facilities. Similar findings have been reported in a study from Kenema district, which showed a reduction in the numbers of pregnant and lactating women accessing services and in Guinea, where there was a 31% reduction in outpatient visits; in both studies, this was attributed to fear of contracting Ebola.^14^ ^15^

Women who accessed care at a health facility were significantly more likely to die and more likely to have a stillbirth during the EVD epidemic. However, our results show that healthcare facilities were in principle ‘ready’ and EmOC was available. Healthcare facilities were able to continue to provide all the components (signal functions) of EmOC with only the availability of assisted vaginal delivery showing a statistically significant reduction. This may be due to government guidance to healthcare providers to avoid practising ‘invasive’ procedures which would increase their exposure to the Ebola virus.^16^ Local by-laws restricting movement between villages may also have limited women's access to healthcare facilities. However, this would have to be considered alongside the Government's requirement for all women to deliver in healthcare facilities rather than in the community.^16^

The increase in mortality for mothers and babies could be explained by two factors: (1) women accessed care late, and/or (2) the quality of the care provided was poor. Timely diagnosis and early intervention is needed to save lives in case of an obstetric emergency. Assisted vaginal delivery, manual removal of retained placenta in case of obstetric haemorrhage, caesarean section, manual vacuum aspiration or curettage in case of haemorrhage associated with miscarriage, and even neonatal resuscitation can all be considered life-saving but are ‘invasive’ and require personal protection for the healthcare provider having to deal with such emergencies. There are anecdotal reports and observations of women being ‘left to deliver on their own’, and it is plausible that healthcare providers were reluctant to intervene early and quickly when this was needed thus not providing safe, timely and effective treatment with a resulting poorer outcome.

## Conclusions

There is no doubt that the Ebola virus epidemic had a devastating effect on Sierra Leone and lessons are being learnt to improve healthcare delivery in the future. This study shows that maternity care was in principle available and continued to be provided. However, the care may in some cases have been provided late by healthcare providers who were afraid of being infected or because women accessed services late. EmOC was available and is potentially life-saving provided complications are recognised and managed early and quickly. During any epidemic—EVD or other—the public need to be confident that healthcare providers can continue to provide both routine and emergency maternity care while at the same time dealing with the effects of the epidemic. Similarly, healthcare providers need to be supported to be able to provide the highest quality of care, being able to distinguish between women infected with EVD who need isolation and/or referral to special treatment centres and women who need EmOC in a timely and safe manner. Healthcare providers need to be able to safely practise with no risk to their or others' personal health. For countries where the EVD epidemic occurred, this requires urgent attention—both for the immediate restoration of routine health services and to be able to ensure continued access to and uptake of high-quality care during future epidemics.
